# Vocal Tremor: Novel Therapeutic Target for Deep Brain Stimulation

**DOI:** 10.3390/brainsci6040048

**Published:** 2016-10-10

**Authors:** Vinod K. Ravikumar, Allen L. Ho, Jonathon J. Parker, Elizabeth Erickson-DiRenzo, Casey H. Halpern

**Affiliations:** Department of Neurosurgery, Stanford University, 300 Pasteur Dr., Edwards Bldg. R-293, Stanford, CA 94305, USA; aho5@stanford.edu (A.L.H.); parkerjo@stanford.edu (J.J.P.); edirenzo@ohns.stanford.edu (E.E.); chalpern@stanford.edu (C.H.H.)

**Keywords:** deep brain stimulation, awake DBS, ventral intermediate nucleus, VIM, dysphonia, essential tremor, essential vocal tremor, EVT, voice, tremulous voice, laryngoscopy, acoustic analysis, microelectrode recording

## Abstract

Tremulous voice is characteristically associated with essential tremor, and is referred to as essential vocal tremor (EVT). Current estimates suggest that up to 40% of individuals diagnosed with essential tremor also present with EVT, which is associated with an impaired quality of life. Traditional EVT treatments have demonstrated limited success in long-term management of symptoms. However, voice tremor has been noted to decrease in patients receiving deep brain stimulation (DBS) with the targeting of thalamic nuclei. In this study, we describe our multidisciplinary procedure for awake, frameless DBS with optimal stimulation targets as well as acoustic analysis and laryngoscopic assessment to quantify tremor reduction. Finally, we investigate the most recent clinical evidence regarding the procedure.

## 1. Introduction

Tremulous voice is a distinguishing feature of many neurologic disorders including Parkinson’s Disease (PD), stroke, orofacial dystonia, spasmodic dysphonia, myasthenia gravis, and progressive supranuclear palsy [1,2,3]. Tremulous voice is characteristically associated with essential tremor (ET), and is referred to as essential vocal tremor (EVT). EVT is thus a manifestation of the oscillatory and rhythmic features of ET in the phonatory apparatus. Current estimates suggest that up to 40% of individuals diagnosed with essential tremor also present with EVT [4]. EVT is characterized by increased vocal effort, particularly during periods of anxiety or stress, causing significant social embarrassment. Patients with severe cases of EVT may feel obliged to reduce social commitments, extricating themselves from employment and hobbies. Though discernable in many forms of vocal interaction, irregularities are most noticeable with sustained phonation of vowels. Thus, EVT may significantly impair quality of life, and patients are in need of a durable solution [5]. 

Tremulous voice is a symptom of laryngeal tremor. The antagonistic contractions of pharyngeal, laryngeal, and/or palatal muscles cause rhythmic oscillations of the laryngeal apparatus at a rate of 4–8 Hz in EVT patients. Such oscillations alter voice pitch and intensity, corresponding to acoustic changes in fundamental frequency (*f*_0_) and amplitude, respectively [5]. Symptoms may be exacerbated by involuntary movements of vocal accessory muscles such as chest wall muscles, abdominal muscles, and the diaphragm. Regularity and intensity of acoustic changes are subject to considerable variability between EVT patients [6].

## 2. Patient Evaluation/Assessment of EVT

The multidisciplinary outpatient evaluation of EVT includes an otolaryngologist specialized in voice disorders as well as a speech language pathologist. Patients will present with debilitating, progressive vocal tremor that is refractory to medication and causing a significant reduction in quality of life. Other tremors such as head or upper extremity tremor may also be present. 

If the patient should present de novo to the neurosurgeon for consideration of deep brain stimulation (DBS) for tremor, normal preoperative DBS work-up and clearance should be completed. The patient must also consult a laryngologist and speech language pathologist for a full tremor work-up. Diagnosis and characterization of EVT is performed by a laryngologist with fiberoptic nasolaryngoscopic examination of the pharynx and larynx, also known as flexible distal-chip laryngoscopy, during tasks that emphasize prolonged phonation [5]. The preoperative examination by the speech language pathologist serves to characterize instrumental and acoustic qualities of the vocal tremor, to develop behavioral strategies for patients to reduce phonatory collision forces at the region of identified pathology. These objective measures also establish a baseline from which to measure intra- and postoperative alterations in voice with DBS [6].

Additional, objective acoustic measures may concomitantly be used to assist with diagnosis [7]. Though transnasal and oral endoscopic approaches are used in EVT diagnosis, the transnasal approach is preferred since it permits a more thorough investigation of the musculature while keeping the patient in a natural posture. Laryngeal tremor may be diagnosed on transnasal endoscopy upon identification of characteristic rhythmic oscillations of the vocal folds during speech. This rhythmic oscillation may be complemented with craniocaudal laryngeal oscillations and/or contractions of the palate as well as the pharynx. Tremor is noticeable both on prolonged vowel phonation as well as during quiet respiration [5,8].

An acoustical examination, comprised of sustained and short sentences as well as sustained vowel sounds, should be utilized to supplement findings and conclusions drawn from a direct laryngeal exam [9]. During the evaluation, it is essential that the patient sustain phonation of vowels since connected speech may conceal the presence or severity of EVT. The rates of *f*_0_ and intensity change are measured, recorded, and used to describe the acoustic characteristics of EVT. Changes in these two rate parameters are typically synchronous, and are the most noticeable features of vocal tremor. Additionally, the magnitude of change in intensity and *f*_0_ may also be utilized in describing EVT acoustic characteristics. Magnitude values are determined by calculating the ranges of both intensity and *f*_0_ oscillations. High voice intensity is characteristic of an ET patient, and successful intervention will likely result in a reduction [10]. Investigation of other acoustic measures such as shimmer, jitter, and harmonic-to-noise ratio, speech rate (syllables per second), and voice aerodynamics (e.g., s-to-z ratio and maximum phonation time) may also be used to preoperatively evaluate patients or assess vocal changes [7]. EVT patients experience increases in shimmer and jitter, as well as a reduction in harmonic-to-noise ratio (voice quality), s-to-z ratio, and maximum phonation time. Therefore, a shift in these parameters can signify reduction of ET symptoms and may be used as markers to verify accurate lead and electrode placement in vocal tremor patients.

## 3. Treatment of EVT

Traditional EVT treatments have demonstrated limited success in long-term management of symptoms. First-line pharmacologic treatment with agents such as primidone and propranolol yield varied outcomes [11]. Alternatively, EVT patients whose tremor originates in the thryroarytenoid and extralaryngeal muscles have received targeted injections of Botulinum A toxin (Botox). Such targeted Botox therapies have demonstrated a significant reduction in tremor amplitude in up to 80% of this subset of patients [12]. However, it should be noted that patients rarely experience complete tremor resolution, despite the transient nature of this treatment and need for periodic injections over the patient’s lifetime. Botox injections may also present with adverse effects including dysphagia, coughing, choking, and breathiness. This side effect profile limits use of the treatment in elderly patients [13]. 

Currently, there are promising avenues for behavioral modification treatment by working with a speech language pathologist [6]. However, it is evident that an enduring and effective treatment for EVT is required. 

## 4. DBS for EVT

Despite shortcomings of pharmacological and behavioral treatments for EVT, Deep Brain Stimulation (DBS) is known to be a safe and reputed method of reducing nonspecific tremor severity for patients who are refractory to medication [7]. However, there remains little research or evidence in the literature that has systematically investigated symptom improvement EVT upon DBS intervention, and many laryngologists remain under-informed regarding the therapeutic potential of DBS [3]. 

In 2002, Sataloff et al. reported the first case report for the application of DBS in treating vocal tremor [14]. The study examined two patients who were implanted with stimulators bilaterally in the ventral intermediate nucleus (Vim) of the thalamus. Voice analysis and strobovideolaryngoscopy revealed elimination of symptoms in one patient and significant decrease in voice tremor in the other [14]. Most studies since that time are comprised of results from small number of participants or are in the form of case reports [15,16,17,18,19].

Voice tremor has been noted to decrease in patients receiving DBS treatment for other indications including dystonia, essential hand tremor, and PD-associated tremor due to the targeting of nearby thalamic nuclei [2,15,20]. For example, DBS targeting the STN for treatment of PD demonstrates improvement in voice tremor symptoms, albeit inconsistent [2]. Patients without Vim targeting did not experience as great a reduction in voice tremor as other regions with tremor [15,16,21].

Of note, patient eligibility and selection should account for potential worsening of gait especially following bilateral Vim DBS implantation, as noted in certain studies [21]. However, gait imbalances can be partially or fully resolved with reprogramming.

## 5. Vim Target for DBS

In 1987, Benabid et al. was first to suggest that tremor symptoms could be alleviated by stimulating the Vim region of the thalamus [22]. The Vim is organized in a somatotopic fashion, with cerebellar afferents from the face positioned most medially, and progressing to hands then feet, laterally. Since the precise location of Vim may be difficult to discern on, microelectrode recording and stereotactic referencing are utilized for physiological mapping and indirect targeting, respectively. The coordinates of the Vim are different for each individual, but as a general rule, direct Vim targeting proceeds 6 mm posterior to the midpoint of the anterior commissure-posterior commissure (AC-PC) and 12–14 mm lateral to the AC-PC line [7]. Microelectrode unit activation occurs more frequently with passive kinesthetic movement of extremity joints, as it passes from the skull entry point caudally to the Vim.

Receptive fields in the thalamus are arranged in a somatotopic map with fields corresponding to the face located medially, upper extremity located ventromedially, and lower extremity located dorsolaterally. As the electrode descends in the customary dorsolateral-to-ventromedial track into the Vim, it will pass leg and subsequently arm kinesthetic receptive fields [6]. Therefore, suppression of voice tremor is contingent on stimulator positioning 1–2 mm medial the typical essential tremor (ET) target [23,24]. 

Since vocal tremor must be continually monitored by a speech language pathologist, surgery is always conducted in the awake state. Tremor can be monitored in regions associated with vocal tremor and which demonstrate tremor symptoms, such as the head and limbs. Before electrode insertion, depth of the target in the Vim is determined. The microelectrode is initially set to a point 15 mm proximal to the target to test and confirm its impedance range. 

The microelectrode is then moved incrementally towards the target, while continuously recording. Single unit recordings when the electrode passes through the thalamus are typically excellent. As the electrode nears the target, macrostimulation is accompanied by meticulous documentation of tremor reduction and careful monitoring for paresthesias. A neurophysiologist will help record kinesthetic responses the corresponding extremity, proximal motor groups, and macrostimulation via the microcannula. Additionally, a speech language pathologist will perform both an intraoperative acoustic assessment and a speech assessment for the patient. 

Accuracy in electrode implantation at an appropriate distance from certain nuclei is contingent on proper identification of surrounding structures. Slightly anterior to the Vim are the ventrooralis posterior and anterior nuclei, which are activated by movement of the contralateral extremities. By contrast, the ventral caudalis nucleus is located posteriorly to the Vim. It can be distinguished from the Vim as it contains a narrow somatotopic area of receptive fields reacting to light touch [25]. Electrode implantation is considered safe if it is sufficiently far from the ventral caudalis nucleus (>2 mm). In ET patients, the DBS electrode is usually targeted to the kinesthetic fields, which are linked to the hands. Successful or near-complete tremor suppression will require macroelectrode stimulation with currents as low as 0.2 mA and microelectrode stimulation with currents up to 100 microAmps. If the electrode is implanted distally from the Vim and proximally to the ventral caudalis nucleus, the patient will experience paresthesia in place of tremor suppression. Thus, successful stimulation will manifest as decreased tremor without any sustained sensation of paresthesia [26]. 

Following successful electrode insertion and verification with macrostimulation and microrecording, the lead should be implanted utilizing the same track. Optimal placement of the electrode will necessitate several passes, introducing a possibility of morbidity. Upon observation of tremor reduction, a Medtronic 3389 DBS electrode, sized to the correct length, may be relayed to the target point along the track. Following test simulations that confirm tremor reduction and rule out the presence of harmful side effects, the lead is secured. This entire process is repeated in the contralateral side for bilateral tremor patients. After one week, the patient returns to have a pulse generator implanted infraclavicularly. Two weeks post-implantation, the patient returns for a programming visit, and will also receive both speech and neurophysiologic analysis. 

## 6. Discussion

EVT is a type of tremulous voice disorder that is highly refractory to treatment, and results in significantly compromised quality of life for patients. Though a decade has passed since DBS had been hypothesized as a therapeutic avenue for patients with EVT, the literature remains sparse with respect to systematic studies. DBS has been proposed as a more permanent treatment modality for EVT patients, but few comprehensive studies exist in the literature investigating DBS use for this particular indication. The first of these studies was performed by Sataloff et al. and utilized objective voice analysis and strobovideolaryngoscopy to evaluate patient outcomes. Both patients in the study experienced significant reductions in vocal tremor, with one patient emerging symptom-free after treatment [14]. Most other studies examining DBS-induced vocal tremor reduction feature patients with an assortment of other pathologies and utilization of differing techniques of intervention [15,16,18,21,27]. Additionally, tremor assessments are not complete and often contain varied time intervals during which recordings were made. Furthermore, qualitative assessment protocols introduce variation between stimulator settings, resulting in more variation in methodology. Moreover, certain tremor evaluations conducted alone, such as subjective speech evaluations without any other examination, have little reliability [7].

In this article, we aim to provide a detailed account of the comprehensive and multidisciplinary use of awake, frameless DBS in treating EVT, beginning with the pre-operative evaluation. Characteristic features of our procedure include designation of the Vim as the neurophysiological target and utilization of real-time physiologic feedback to track changes in vocal tremor while in the operating room. Utilization of a frameless system provides added benefits in comparison to the stereotactic frame equivalent, including ease of intraoperative neurological examinations, improved patient comfort, greater accuracy, as well as real-time electrode tracking by synthesizing multiple information sources. These benefits are demonstrated in the clinical setting without compromising either stability or accuracy [28].

Notably, our procedure is successful in reducing symptoms of EVT without minimizing the positive effects of DBS on tremor in the concurrent extremities. DBS intervention results in an increased harmonic-to-noise ratio (improved voice quality) and decreased *f*_0_ [7]. More investigation is required to uncover the prime electrode targets to concurrently treat multiple tremor types. Moreover, Vogel et al. found that optimal stimulator settings, including the Pitch Floors/Ceilings and Time Steps, are superior in function when compared to baseline, clinical settings [7]. However, we believe that this effort will help influence and inspire the development of new treatments for this previously refractory group of patients. 

Despite their limitations, these studies exhibit positive results of DBS treatment, including decreases in both tremor severity and the rate of F0 modulation [6,15]. It is imperative that further prospective investigations are conducted to validate the positive effects of DBS in EVT patients. Furthermore, it is important to distinguish the difference between successful tremor reduction and speech outcomes. While DBS has demonstrated promise and consistent success in reducing tremor, the correlation between tremor decrease and speech improvement is unclear [7,29]. A recent study examining the use of DBS for EVT in a different brain region found that vocal tremor treatment significantly reduced symptoms in only half of the patient cohort [30]. This suggests the possibility of vocal tremor symptoms that originate in other parts of the body, from sources outside of the direct phonatory apparatus. Hence, careful evaluation of the source of tremor by a trained otolaryngologist is essential. 

Future studies should incorporate larger patient cohorts studied over long periods of time in order to truly examine efficacy and complications of DBS as a treatment for vocal tremor. The focal, reportable metric in these studies will be a complete voice and larynx examination pre-, intra-, and post-operatively, which will help in the production of evidence-based guidelines. This examination should incorporate nasal endoscopy for direct laryngeal visualization as well as vocal analysis, such as aerodynamics, review by both patient and physician, and acoustics. Results from such studies will have the potential to shape future therapies for this group of medication-refractory patients. 

## 7. Conclusions

In this study, we describe our multidisciplinary procedure for awake, frameless DBS with optimal stimulation targets as well as acoustic analysis and laryngoscopic assessment to quantify tremor reduction. 
